# Characterization of highly efficient heavy-ion mutagenesis in *Arabidopsis thaliana*

**DOI:** 10.1186/1471-2229-11-161

**Published:** 2011-11-15

**Authors:** Yusuke Kazama, Tomonari Hirano, Hiroyuki Saito, Yang Liu, Sumie Ohbu, Yoriko Hayashi, Tomoko Abe

**Affiliations:** 1 RIKEN Nishina Center, 2-1 Hirosawa, Wako, Saitama 351-0198, Japan; 2 RIKEN Innovation Center, 2-1 Hirosawa, Wako, Saitama 351-0198, Japan

## Abstract

**Background:**

Heavy-ion mutagenesis is recognised as a powerful technology to generate new mutants, especially in higher plants. Heavy-ion beams show high linear energy transfer (LET) and thus more effectively induce DNA double-strand breaks than other mutagenic techniques. Previously, we determined the most effective heavy-ion LET (LET_max_: 30.0 keV μm^-1^) for *Arabidopsis *mutagenesis by analysing the effect of LET on mutation induction. However, the molecular structure of mutated DNA induced by heavy ions with LET_max _remains unclear. Knowledge of the structure of mutated DNA will contribute to the effective exploitation of heavy-ion beam mutagenesis.

**Results:**

Dry *Arabidopsis thaliana *seeds were irradiated with carbon (C) ions with LET_max _at a dose of 400 Gy and with LET of 22.5 keV μm^-1 ^at doses of 250 Gy or 450 Gy. The effects on mutation frequency and alteration of DNA structure were compared. To characterise the structure of mutated DNA, we screened the well-characterised mutants *elongated hypocotyls *(*hy*) and *glabrous *(*gl*) and identified mutated DNA among the resulting mutants by high-resolution melting curve, PCR and sequencing analyses. The mutation frequency induced by C ions with LET_max _was two-fold higher than that with 22.5 keV μm^-1 ^and similar to the mutation frequency previously induced by ethyl methane sulfonate. We identified the structure of 22 mutated DNAs. Over 80% of the mutations caused by C ions with both LETs were base substitutions or deletions/insertions of less than 100 bp. The other mutations involved large rearrangements.

**Conclusions:**

The C ions with LET_max _showed high mutation efficiency and predominantly induced base substitutions or small deletions/insertions, most of which were null mutations. These small alterations can be determined by single-nucleotide polymorphism (SNP) detection systems. Therefore, C ions with LET_max _might be useful as a highly efficient reverse genetic system in conjunction with SNP detection systems, and will be beneficial for forward genetics and plant breeding.

## Background

Mutation induction is a powerful tool for analysis of gene function and breeding. Among the mutagens that have been used to induce mutations, chemical mutagens such as ethyl methane sulfonate (EMS), or ionising radiation such as X-rays or γ-rays, have been especially popular in plant science. EMS can produce point mutations, mainly G/C-to-A/T transitions, with high frequency [1,2]. Such point mutations are easily detected by mutation-detection systems such as the CEL1 nuclease assay or high-resolution melting curve (HRM) analysis [3,4]. In combination with a single-nucleotide polymorphism (SNP) detection system, EMS-mediated mutagenesis is a powerful reverse genetics approach, called Targeted Induced Local Lesions in Genomes (TILLING) [5-9]. Because of its mutation-inducing property, EMS is also very useful for producing leaky alleles in forward genetics. By contrast, X-rays and γ-rays induce DNA damage relatively randomly and cause many types of mutations including base substitutions, deletions and chromosomal alterations [10,11]. Although X-rays and γ-rays are suitable for production of null mutations, the mutation frequency induced by X-rays and γ-rays is lower than that obtained by EMS.

Heavy-ion beams are accepted as a novel powerful mutagen because they are able to induce mutations with high frequency at a relatively low dose at which virtually all plants survive, and they induce a broad spectrum of phenotypes without affecting other plant characteristics [12,13]. These characteristics of heavy-ion beams are advantageous for mutation breeding. Over 30 plant cultivars have been bred with the aid of heavy-ion beams in Japan [14,15]. Heavy-ion beams comprise accelerated ions produced by an ion accelerator such as a cyclotron or synchrotron. A noted physical characteristic of a heavy-ion beam is that the accelerated particles densely deposit their energy in a localized region along the particle path. This is strikingly different from γ-rays and X-rays, which sparsely deposit their energy in a large targeted volume. The degree of locally deposited energy is represented by the linear energy transfer (LET; the energy transferred per unit length, keV μm^-1^). Whereas the LETs of γ-rays and X-rays are 0.2 and 2.0 keV μm^-1^, respectively, the LET of a heavy-ion beam for use in biological research ranges from 22.5 keV μm^-1 ^to 4000 keV μm^-1 ^in the RIKEN RI-beam factory (RIBF) [16]. It is well known that high-LET radiation shows stronger biological effects than low-LET radiation. The LET of a heavy-ion beam is selectable by ion species, and depends on the characteristics of the ion with respect to electrical charge and velocity. When a high LET is required, a heavier and highly charged ion with a low velocity is selected.

Based on radiobiological considerations, it has been suggested that heavy-ion beams predominantly induce double-strand breaks (DSBs) [17,18]. A high yield of DSBs after heavy-ion beam irradiation was revealed by experiments on both animal and plant cells [19,20]. Therefore, significant DNA damage is likely to be caused by heavy-ion irradiation, although sequencing analysis of heavy-ion-induced DNA alterations is limited. Shikazono et al. reported that about half of the mutations induced by carbon (C) ions with LET of 101-124 keV μm^-1 ^were small alterations, including base substitutions and comparatively small insertions/deletions (under 100 bp), whereas the other half were rearrangements such as translocations, inversions, and comparatively large insertions/deletions (over 100 bp) [21]. These results indicate that heavy-ion irradiation induces a broad range of mutations.

In a previous study, we found that the LET value affects the albino-mutant incidence in the M_2 _generation and that 30 keV μm^-1 ^is the most effective LET value in *Arabidopsis thaliana *mutagenesis [22]. This high-efficiency LET (termed LET_max_) should be beneficial not only for forward genetics and breeding, but also for reverse genetics [23]. However, the mechanisms that contribute to the efficient mutagenesis of LET_max _irradiation are still unclear. Because mutagens such as EMS, γ-rays, and heavy-ion beams must be chosen appropriately depending on the experimental purpose or target genes, it is also important to know the nature of mutations induced by C ions with LET_max_. In the present study, we investigated the relationship between mutation induction and parameters of heavy-ion irradiation, which comprised the number of irradiated ion particles and LET. We also determined mutations in knock-out mutants induced by C-ion irradiation with LET_max _in *A. thaliana*, as a first step to characterize the nature of C-ion induced mutations.

## Results

### Analysis of particle number and LET effects on mutation frequency

**Figure 1:**
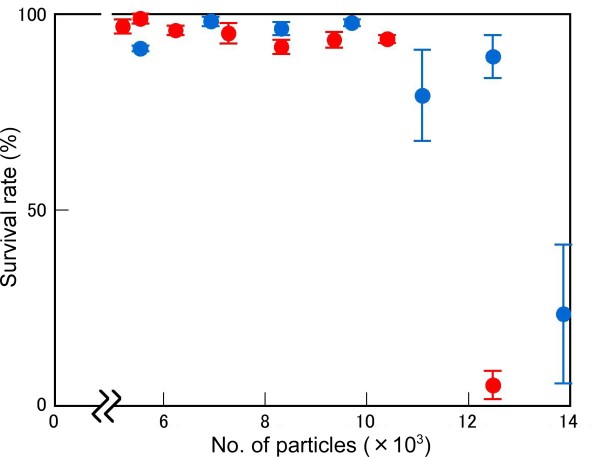
**LET-dependent effect of C ions on survival**. Survival (%) was recorded 1 month after sowing C-ion irradiated seeds. Blue and red circles indicate 22.5 keV μm^-1 ^and 30.0 keV μm^-1 ^LET, respectively.

**Figure 2:**
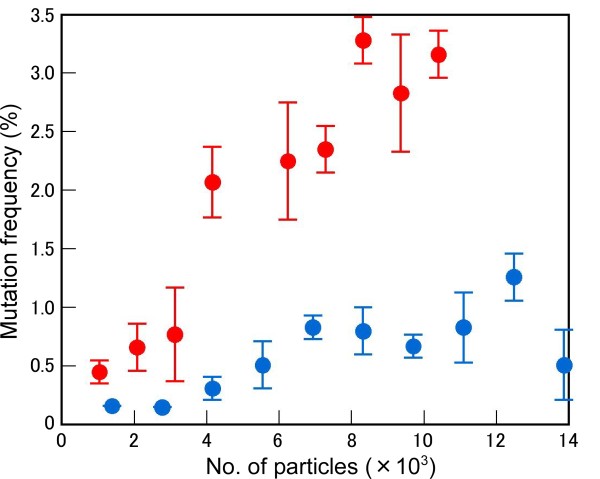
**LET- and particle number-dependent effects of C ions on mutation induction**. Mutation frequencies were investigated in the M_2 _generation by counting the number of albino mutants 8 d after the onset of germination. Blue and red circles indicate 22.5 keV μm^-1 ^and 30.0 keV μm^-1 ^LET, respectively.

To achieve increased mutation efficiency with heavy-ion irradiation, the effects of both the number of irradiated ion particles and the LET value should be studied. The number of ion particles could determine the number of DSBs per cell nucleus, while the LET value might affect the efficiency of DSB induction (see Discussion). In a previous study, we found that C-ion irradiation with LET_max _induced a three-fold higher mutation frequency than that with 22.5 keV μm^-1 ^[22]. To confirm the effect of both LET and particle number more precisely, C-ion irradiation was applied at doses that ranged from 50 to 600 Gy; the survival percentage in the M_1 _generation and albino incidence in the M_2 _generation were measured. The number of ion particles per cell nucleus was calculated based on the assumptions that seeds have a specific density of 1 and the size of the nucleus is 100 μm^2 ^(see Methods). The dose (in Gy) is proportional to the LET (in keV μm^-1^) and the number of irradiated particles. The effect of C ions with LET of 30.0 keV μm^-1 ^on survival percentage was greater than that at LET of 22.5 keV μm^-1 ^(Figure 1). For C ions with LET of 30.0 keV μm^-1^, about 12,500 particles per 100 μm^2 ^were needed to cause lethality, whereas at 22.5 keV μm^-1 ^over 14,000 particles per 100 μm^2 ^were required. The LET value had a more striking effect on the mutation frequency in the M_2 _generation than the M_1 _generation (Figure 2). Carbon ions with LET of 30.0 keV μm^-1 ^produced a 3.28% albino incidence at the most effective particle number (8,320 per 100 μm^2^). By contrast, C ions with LET of 22.5 keV μm^-1 ^produced only a 1.26% albino incidence, even at the most effective particle number (12,480 per 100 μm^2^). The difference in LET effect on mutation frequency between 22.5 keV μm^-1 ^and 30.0 keV μm^-1 ^was obvious, especially under irradiation with over 4,000 particles, which indicated that the particle number is also important to obtain a high mutation frequency. These findings indicate that C ions with LET of 30.0 keV μm^-1 ^have a different mutational effect from those with 22.5 keV μm^-1 ^and raise the question as to what DNA alterations are caused by these irradiation conditions.

### Confirmation of mutation efficiency of C ions with LET_max_

The *elongated hypocotyl *(*hy*) and *glabrous *(*gl*) mutants were screened in the M_2 _generation after C-ion irradiation under three conditions: LET of 22.5 keV μm^-1 ^at a dose of 250 Gy (6,933 per 100 μm^2^), 22.5 keV μm^-1 ^at a dose of 450 Gy (12,480 per 100 μm^2^), and 30.0 keV μm^-1 ^at a dose of 400 Gy (8,320 per 100 μm^2^). Mutation frequencies and the structure of mutated DNAs in these conditions were compared. The *hy *and *gl *mutants are well characterised and the genes responsible for the respective phenotypes have been determined [24-31]. Consequently, these mutants have been used previously for similar mutated DNA analyses [21,32,33]. Screening of 29,595 M_2 _plants revealed that 23 mutants were induced by C ions with LET of 30.0 keV μm^-1 ^at a dose of 400 Gy. The mutation frequency with 30.0 keV μm^-1 ^was approximately two-fold higher than that in the other irradiation conditions (Table 1). These results support the preceding data in which C ions with LET of 30.0 keV μm^-1 ^were more effective for mutation induction than those with 22.5 keV μm^-1^.

**Table 1:** Frequencies of *hy *and *gl *mutants among plants irradiated with C ions

LET (keV μm^-1^)	Dose (Gy)	No. of M_1 _plants	No. of M_2 _plants	No. of mutants (mutation frequency (‰)*)
22.5	250	2,024	11,662	5 (0.43)
22.5	450	1,710	16,103	5 (0.31)
30.0	400	3,056	29,595	23 (0.78)

*Total number of isolated *hy *and *gl *mutants

Mutation frequency = no. of mutants/no. of M_2 _plants × 10^2^

### Characterisation of mutated DNA structure caused by C-ion irradiation

To investigate the structure of mutated DNA in the isolated mutants caused by C-ion irradiation, DNA from the isolated mutants was subjected to HRM, PCR, and sequencing analyses using primers specific for the genes responsible for the *hy *and *gl *phenotypes (see Methods). Among the 33 *hy *and *gl *mutants isolated, 18 independent mutant lines were identified. This is because mutants isolated from the same batch were thought to have originated from the same M_1 _plants. To confirm that all mutants classified in the same mutant line had an identical DNA mutation, all of the mutants derived from the same batch were confirmed by PCR and sequencing analysis. Because the number of identified mutant lines was limited, the following mutants were also included in the characterisation of the DNA mutations: *altered meristem program *(*amp*) *1, pinoid *(*pid*) *1*, and *yellow variegated *(*var*) *2 *(see Methods) [34-36].

The identified DNA mutations are listed in Table 2. In total, 22 mutations were identified. Mutations of 17 of the 18 independent *hy *and *gl *mutant lines were determined successfully. In addition, two mutations in the *AMP1 *gene, two mutations in the *PID1 *gene, and one mutation in the *VAR2 *gene were identified. The C-ion-induced mutations consisted of base substitutions, deletions, insertions, and translocations. Of the 22 alleles, only four showed rearrangements, including translocations and a large deletion; these were detected in high-dose irradiated mutants (400 Gy and 450 Gy). Eighteen mutants had base substitutions or deletions/insertions less than 100 bp (Tables 2 and 3). Of the four alleles with a base substitution, three were transversions and one was a transition. Among these, only one allele (C-27-gl1) had a missense mutation (D→N), whereas the other alleles had nonsense mutations that resulted in production of C-terminally truncated proteins. In total, 21 alleles were null mutants. Whether the C-27-gl1 allele was a null mutation was not elucidated, although the phenotype of the C-27-gl1 mutant was similar to that of a null mutant of *GL1 *(data not shown). The size and type of mutations induced by 22.5 keV μm^-1 ^and 30.0 keV μm^-1 ^LET did not differ. These results indicated that C ions with LETs of 22.5 or 30.0 keV μm^-1 ^mainly caused small alterations and that most of the induced mutants were null mutants.

**Table 2:** Mutations induced by C-ion irradiation

LET (keV μm^-1^)	Dose (Gy)	Allele	Mutated gene	Type of mutation*	Size** (bp)	Position
22.5	250	C-27-gl1	*GL1*	BS	G→A	Chr.5: 10,363,437
		C-45-hy1	*HY4*	Del	2	Chr.4: 5,724,273-74
		C-48-amp1	*AMP1*	BS	A→T	Chr.3: 20,255,432
		C-55-hy1	*HY4*	Del	3	Chr.4: 5,725,528-30
		C-142-hy1	*HY3*	Del	1	Chr.2: 8,141,902
		C-162-gl1	*TTG1*	Del	51	Chr.5: 8,371,718-68

	450	C(450)-100-gl1	*TTG1*	Del	1	Chr.5: 8,372,723
		C(450)-124-hy1	*HY3*	BS	G→T	Chr.2: 8,143,452
		C(450)-135-hy1	*HY1*	Del	36	Chr.2: 11,342,207-42
		C(450)-139-pid1	*PID1*	RTL		See Figure 3
		C(450)-150-pid1	*PID1*	Ins	1	Chr.2: 14,590,504
		C(450)-154-hy1	*HY4*	Del	32,335	Chr.4: 5,697,598-730,031

30.0	400	C30-8-gl1		NM		
		C30-9-gl2	*GL2*	Del	1	Chr.1: 30,038,621
		C30-39-hy1	*HY2*	Del	5	Chr.3: 2,805,174-78
		C30-73-gl1	*TTG1*	CR		See Figure 3
		C30-74-hy1	*HY4*	Del	2	Chr.4: 5,725,263-64
		C30-106-gl1	*GL2*	Del	1	Chr.1: 30,039,697
		C30-108-hy1	*HY4*	RTL		See Figure 3
		C30-148-amp1	*AMP1*	Del	1	Chr.3: 20,257,195
		C30-155-hy1	*HY2*	Del	4	Chr.3: 2,804,929-32
		C30-252-gl1	*GL2*	BS	C→A	Chr.1: 30,040,486
		C30-273-var1	*VAR2*	Del	23	Chr.2: 13,175,250-72

* BS, Base substitution; CR, complex rearrangement; Del, deletion; Ins, insertion; RTL, reciprocal translocation; TL, translocation, NM, no mutation in the genes sequenced in this study.

**For base substitutions, changed bases are specified.

**Table 3:** DNA insertion or deletion mutations induced by C-ion irradiation

Allele	Sequence change	
	Original sequence	Mutant sequence
C-45-hy1	TCTGGTTCTG**T**a**t**CTGGTTGTGGT	TCTGGTTCTG **T **CTGGTTGTGGT
C-55-hy1	GGCCGGACTGgatATCCGTTGGTC	GGCCGGACTG ATCCGTTGGTC
C-142-hy1	GATGCGATTCAcTCGCTCCAGCT	GATGCGATTCA TCGCTCCAGCT
C-162-gl1	AGTGGT**CTT**cttcgc•••ctc**ctt**AGAGTT	AGTGGT **CTT **AGAGTT

C(450)-100-gl1	CATGGATAATTcAGCTCCAGATT	CATGGATAATT AGCTCCAGATT
C(450)-135-hy1	AAAAC**TCA**caa•••cagt**tca**AGAG	AAAAC **TCA **AGAG
C(450)-150-pid1	AACTCCGTTCACCGCGAC	AACTCCGTT ***T ***CACCGCGAC
C(450)-154-hy1	AACTAAaccgta•••ggtatgGTTCA	AACTAA GTTCA

C30-9-gl2	TGCAGGCTA**Tt**CAAAGAGACA	TGCAGGCTA **T **CAAAGAGACA
C30-39-hy1	CATTGAAC**A**ggaa**a**TCCCTTAGC	CATTGAAC **A **TCCCTTAGC
C30-74-hy1	TTCTTTCTccACACTTGC	TTCTTTCT ACACTTGC
C30-106-gl1	AGTGTAC**Tt**CGTGAGAAG	AGTGTAC **T **CGTGAGAAG
C30-148-amp1	CTTGGGAAGaGGAGCAATT	CTTGGGAAG GGAGCAATT
C30-155-hy1	TGACATGG**C**gag**c**ACAAAAGGT	TGACATGG **C **ACAAAAGGT
C30-273-var1	GGTTT**GT**tcctt•••gtctg**gt**GGTGG	GGTTT **GT **GGTGG

Deleted sequences are indicated in lower case. Overlapping sequences found in deletion sites are highlighted in bold. Inserted sequence is highlighted in bold and *Italic*.

**Figure 3:**
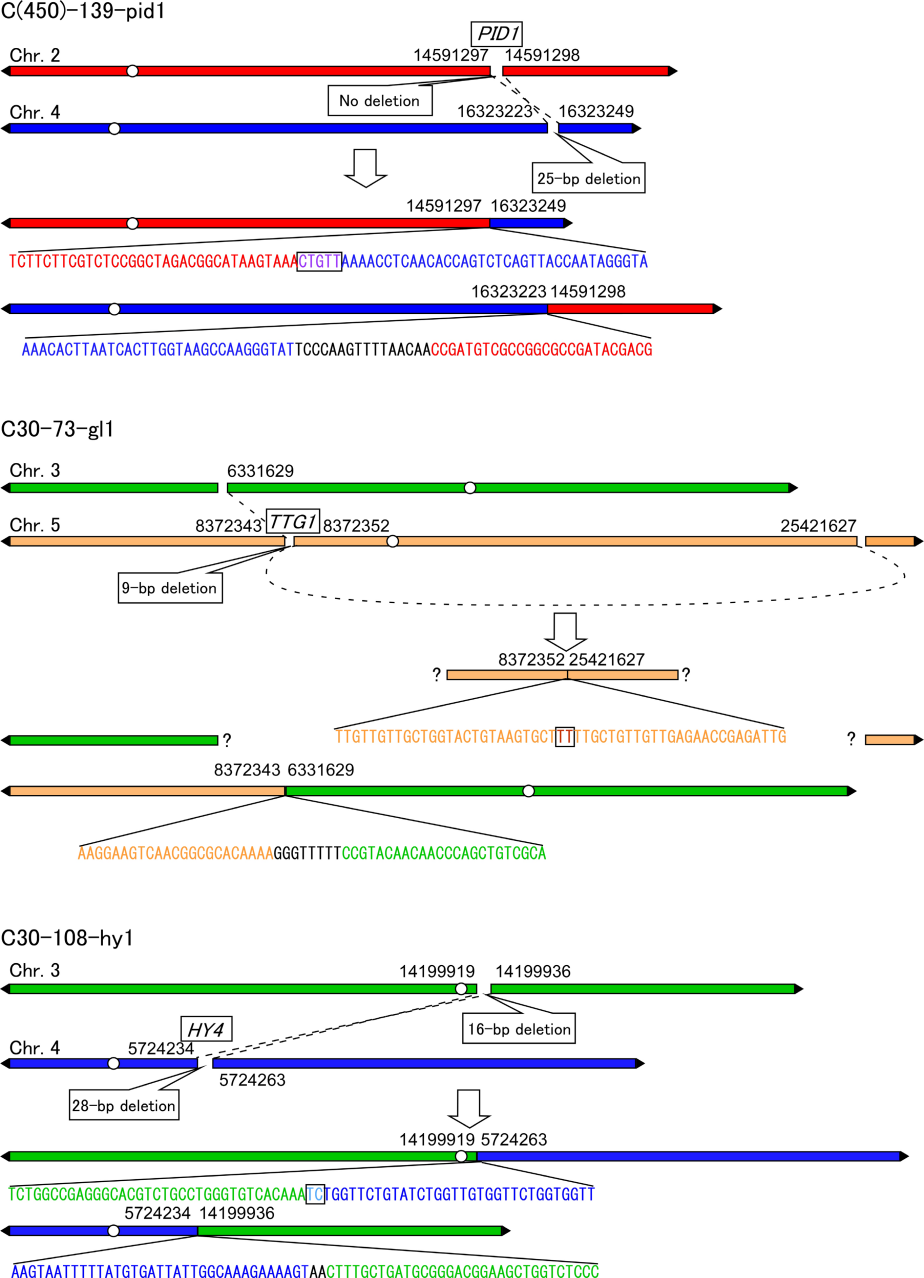
**Schematic representation of rearrangements identified in this study**. The DNA sequences of rearranged chromosomal regions confirmed by PCR and sequencing are shown. C(450)-139-pid1 and C30-108-hy1 have reciprocal translocations between chromosomes 2 and 4, and chromosomes 3 and 4, respectively. In the C30-73-gl1 mutant, the 5' end of the *TTG1 *gene was connected to chromosome 3, while the 3' end of the gene was connected to a region of chromosome 5. White circles indicate centromeres. Filler DNAs are indicated by black letters. Overlapping sequences are boxed. Mutation positions are numbered according to the TAIR9 annotation.

Two reciprocal translocations and one complex rearrangement were detected (Figure 3). For the complex rearrangement (C30-73-gl1), only one breakpoint at the *TTG1 *gene was detected by TAIL-PCR. The other possible irradiation-induced breakpoints in the mutant could not be determined by any PCR analysis. However, five breakpoints were identified successfully in the mutants with rearrangements. Of the five breakpoints, four contained deletions (ranging from 9 to 28 bp), one had no deletion, and none had duplications (Figure 3). These five breakpoints were repaired, which resulted in six rejoined sites. Half of the rejoined sites showed short regions of sequence homology (microhomology; 2-5 bp), whereas the other half had inserted DNA fragments (3-16 bp), termed filler DNA [37]. Fourteen rejoining sites of simple deletions are listed in Table 3. Eight of these rejoined sites showed 1-3 bp microhomology.

## Discussion

In this study, we characterised the mutation frequencies and structure of mutated DNAs in knock-out mutants caused by C-ion irradiation with LET of 22.5 keV μm^-1 ^or 30.0 keV μm^-1 ^(LET_max_). The mutation frequency for C-ions with LET of 30.0 keV μm^-1 ^was higher than that for C ions at 22.5 keV μm^-1^, as indicated by frequencies of albino mutants (Figure 2) and *hy *and *gl *mutants (Table 1). Although the number of mutants identified is too small for accurate statistical analysis, the *hy *and *gl *mutation frequencies induced by C ions with LET of 30 keV μm^-1 ^are similar to that induced by EMS (0.87‰) and 2.5-fold higher than that induced by X-rays (0.32‰) [38]. By contrast, the structure of mutated DNA induced by C ions with LET of 22.5 keV μm^-1 ^or 30.0 keV μm^-1 ^was almost identical. In both cases, over 80% of the determined mutations were small alterations and the remainder were rearrangements (Table 2). The proportion of large genetic alterations that followed repair of irradiation-induced damage might be higher than that observed in this study because a large alteration affecting an essential gene might not be transmitted to the M_2 _generation [39]. Twenty-one of the 22 mutated DNAs characterised were null mutations since these mutations cause a frameshift or a nonsense mutation. The actual proportion of base substitutions induced by irradiation might be higher than that detected in this study, because some might represent silent mutations that could not be identified in the current screening. Base substitutions and small deletions/insertions were detectable by the CEL1 nuclease assay or HRM analysis. Indeed, most of the mutations identified in this study were determined by HRM (see Methods). From our results, we suggest that C-ion irradiation at LET_max _can be used for effective TILLING to obtain null mutants.

Mutagens must be selected according to the experimental purpose or target genes. Carbon-ion irradiation has potential advantages for several aspects of mutagenesis. First, C-ion irradiation is more practical to administer than EMS. EMS treatment of tissues or plantlets is sometimes time-consuming because of its penetration capability. On the other hand, the irradiation times required with C-ions are short; only a few seconds irradiation is needed for imbibed seeds, tissues, and plantlets, or a few minutes for dry seeds. Indeed, by irradiation of tissue cultures or plantlets with C-ion beams, over 20 novel cultivars in diverse plant species have been produced. Second, C-ion irradiation may be advantageous in the induction of truncation mutants rather than generation of allelic series, because our data indicated that small indel mutations occurred more frequently than base-change mutations. To generate an allelic series, EMS induction of base substitutions is useful. Finally, C-ion irradiation might have potential to induce deletions with desired sizes by selection of an appropriate LET value (see below). To clarify the beneficial characteristics of mutation induction by C-ion irradiation, whole-genome investigation of mutations such as missense and silent mutations is needed.

The proportion of small alterations, such as base substitutions or small deletions/insertions and rearrangements, induced by C ions with LET of 30.0 keV μm^-1 ^or 22.5 keV μm^-1 ^is more similar to that induced by low-LET radiation (electrons) than that induced by C ions at 101-124 keV μm^-1 ^LET (Table 4). Similar proportions were reported in irradiation experiments on transgenic mice, in which C ions with LET of 21.3 keV μm^-1 ^preferentially induced small alterations (68%), as did γ-rays (71%) [40]. Suzuki et al. reported that the proportions of large and small deletions differed between C ions with LETs of 39 keV μm^-1 ^and 124 keV μm^-1 ^in an irradiation experiment on human cells [41]. These data indicate that C ions with moderate LET (around 30 keV μm^-1^) might have different effects on DNA alterations from C ions with LET of 101-124 keV μm^-1^. However, the structure of breakpoints was similar between the current study (22.5 keV μm^-1 ^or 30.0 keV μm^-1^) and a previous study (101-124 keV μm^-1^) [21]. In the present study, five of the six breakpoints of rearrangements had deletions, whereas no breakpoint contained a duplication (Figure 3). A previous study revealed that the breakpoints induced by C ions with LET of 101-124 keV μm^-1 ^preferentially have deletions (11 out of 17), whereas the breakpoints induced by electrons tend to have duplications (6 out of 8) [21]. These results imply that the process of DSB production and repair after irradiation with C ions with LETs of 22.5 keV μm^-1 ^or 30.0 keV μm^-1 ^might be similar to those with 101-124 keV μm^-1^. Therefore, 30.0 keV μm^-1 ^appears to represent a moderate LET between low-LET radiation and 101-124 keV μm^-1^.

**Table 4:** Classification of mutations induced by electron and C-ion irradiation

Radiation	Small alterations* (%)	Rearrangements* (%)	Reference
Electron (0.2 keV μm^-1^)	9 (75.0)	3 (25.0)	[21]
C-ion (22.5 keV μm^-1^)	10 (83.3)	2 (16.7)	This study
C-ion (30.0 keV μm^-1^)	8 (80.0)	2 (20.0)	This study
C-ion (101-124 keV μm^-1^)	14 (48.3)	15 (51.7)	[21]

* Small alterations comprise 1-100 bp deletions/insertions and base substitutions. Rearrangements comprise deletions/insertions exceeding 100 bp, translocations, reciprocal translocations and inversions.

Previously, we had no clear answer to the question of why C ions with 30 keV μm^-1 ^LET can induce a higher mutation frequency than other LET values. The current study showed no difference in the structure of mutated DNAs between C-ion irradiation at 22.5 keV μm^-1 ^and 30 keV μm^-1^. Thus, the DSB repair process in both conditions might be the same, although the possibility of existence of a LET-dependent DNA repair pathway cannot be excluded. One possible explanation for the difference in mutation frequencies between these irradiation conditions is the difference in the efficiency of DSB production. Although the number of irradiated particles with LET of 22.5 keV μm^-1 ^was larger than that with 30.0 keV μm^-1^, 22.5 keV μm^-1 ^did not induce a higher mutation frequency than 30.0 keV μm^-1 ^(Figure 2). Therefore, it is likely that 30.0 keV μm^-1 ^C ions induced DSBs more effectively than 22.5 keV μm^-1 ^C ions. With regard to C ions with LET values exceeding 30.0 keV μm^-1^, the mutation frequency was not higher than that of LET_max _(30 keV μm^-1^) [22]. Under these irradiation conditions, the particle number might not be sufficient to produce the same mutation effect as that with LET of 30 keV μm^-1^. For example, with irradiation of 4,000 particles per 100 μm^2 ^at 61.5 keV μm^-1^, the absorbed dose reached about 400 Gy, at which point the M_1 _plants could not survive [22]. Collectively, our results indicate that both an appropriate LET value and an adequate particle number might be needed to obtain the highest mutation efficiency with heavy-ion beam irradiation.

Alternatively, the difference in DSB qualities might be a possible explanation for the LET-dependent difference in mutation induction efficiency between 22.5 keV μm^-1 ^and 30.0 keV μm^-1^. Monte Carlo calculations indicate that high-LET radiation induces a higher fraction of complex DSBs than low-LET radiation [42,43] and complex DSBs are difficult to repair [44,45]. The data from the Monte Carlo calculations are in good accordance with experimental measurements of the higher yield of short DNA fragments after high-LET irradiation in both animals and plants [20,46]. Therefore, it is likely that the quality of DSBs induced by C ions with LET of 30.0 keV μm^-1 ^might be slightly more complex than that induced by C ions with LET of 22.5 keV μm^-1^, and that the complex DSBs might be difficult to repair, although more-detailed theoretical and experimental data with LETs around 30.0 keV μm^-1 ^are required to clarify this hypothesis.

The current data indicate that the structures of mutated DNA caused by C-ion irradiation with LETs of 22.5 keV μm^-1 ^or 30.0 keV μm^-1 ^differed from those induced by C-ion irradiation with LET of 101-124 keV μm^-1^. In *Mesorhizobium loti*, irradiation by iron ions (LET 640 keV μm^-1^) induced larger deletions compared with deletions induced by C ions (LET 23 keV μm^-1^) [47]. These results raise the possibility that the structure of mutated DNA might be controlled by selection of an appropriate LET value. To achieve this, further experimental data under irradiation conditions with different LETs is needed as well as theoretical analysis of the quality of DSBs.

## Conclusions

C ions at LET_max _showed higher mutation efficiency than those with LET of 22.5 keV μm^-1^, with an efficiency that appears similar to that with EMS. To achieve such efficiency with heavy-ion beam irradiation, both LET and particle number must be optimised. In *Arabidopsis*, 30.0 keV μm^-1 ^at 400 Gy (8,320 per 100 μm^2^) was the most effective dose. C ions at LET_max _and 22.5 keV μm^-1 ^predominantly induced null mutations. Over 80% of the null mutations were base substitutions or small deletions/insertions, which can be detected by SNP detection systems such as the CEL1 nuclease assay or HRM analysis. It is concluded that C ions with LET of 30.0 keV μm^-1 ^might be suitable as a powerful TILLING technology in conjunction with a SNP detection system to produce null mutants.

## Methods

### Irradiation treatment

Dry seeds of *A. thaliana *ecotype Columbia (Col-0) were packed in a plastic bag to obtain a monolayer of seeds. The seeds were irradiated with ^12^C^6+ ^ions (22.5 keV μm^-1 ^or 30.0 keV μm^-1 ^LET) with a dose range of 50 Gy to 500 Gy using the E5 beam line in the RIKEN RI-beam factory. The ions were accelerated up to 1.62 GeV, at which the LET value of the ^12^C^6+ ^ions was 22.5 keV μm^-1^. The LET value of the ^12^C^6+ ^ions was adjusted to 30.0 keV μm^-1 ^by reducing the velocity of the ions. To reduce the ion velocity, the ions were passed through a combination of absorbers [48]. All LET values were calculated behind the seeds. The irradiated M_1 _seeds were surface-sterilised by dipping in 1% sodium hypochlorite for 10 min, washed five times with sterilised water, and incubated on 0.7% agar-containing Murashige and Skoog (MS) medium supplemented with MS vitamins and 3% sucrose at 4°C in the dark for 4 d to induce vernalisation. Subsequently, the seeds were incubated at 22°C under long-day conditions (16 h light, 8 h dark). Seedlings that developed true leaves were transplanted into plastic trays (13 × 9 cm^2^) that contained soil. Eleven seedlings were planted in each tray and grown at 22°C under long-day conditions in a greenhouse. The M_2 _seeds were collected from all plants in each tray and were treated as one batch.

### Analysis of particle effect on plant survival and DNA mutation

Measurement of percentage survival of irradiated M_1 _seeds and albino incidence in the M_2 _generation was performed as described previously [22]. At least three independent experiments at different doses of irradiation were carried out for each LET value. To estimate the number of particles per cell nucleus, the number of particles at each dose was calculated for a water area of 100 μm^2 ^with a specific gravity of 1, as described previously [49].

### Mutant screening and identification of mutated genes

From the M_2 _generation, *elongated hypocotyl *(*hy*) and *glabrous *(*gl*) mutants were screened by germination of the M_2 _seeds on MS agar medium. M_2 _plants that showed the *hy *and *gl *phenotypes were isolated. Genomic DNA was purified from the isolated mutant and wild-type plants four weeks after germination using the DNeasy Plant Mini Kit (QIAGEN, Hilden, Germany). The purified DNAs were subjected to HRM analysis using primers specific for the putative mutated genes (*HY1, HY2, HY3*, and *HY4 *for the *hy *mutants; *GL1, GL2*, and *TTG1 *for the *gl *mutants; see Additional file 1). HRM analysis was performed on a LightCycler 480s (Roche Diagnostics, Penzberg, Germany) in a reaction mixture that contained 10 ng wild-type DNA, 10 ng mutant DNA, 0.5 mM of each primer, and 3 mM MgCl_2 _in the LightCycler 480 High Resolution Melting Master containing ResoLight dye (Roche Diagnostics) adjusted to a total volume of 10 μl with PCR-grade water. The reaction conditions comprised an activation step at 95°C for 10 min followed by 50 cycles of 95°C for 10 s, a touchdown of 65°C to 55°C for 10 s (0.5°C cycle^-1^), and 72°C for 10 s. Before the HRM step, the products were heated to 95°C for 1 min and frozen to 40°C for 1 min. HRM analysis was carried out over the range from 65°C to 95°C, rising at 4.4°C s^-1 ^with 25 acquisitions per degree. All reactions were performed in replicate (duplicate or triplicate) in 96-well plates. When a positive signal was identified, the amplified fragment was sequenced using the Big Dye Terminator v. 3.1 Cycle Sequencing Kit (Applied Biosystems) and a 3730xl DNA Analyser (Applied Biosystems) with the same primers as those used for HRM analysis. When the whole or part of the coding region could not be amplified, flanking sequence analysis using TAIL-PCR was performed [50]. Primers used for flanking sequence analysis are listed in Additional file 2. Because of the limited number of *hy *and *gl *mutant lines identified, mutations induced in three additional well-characterised morphological mutants, namely *altered meristem program *(*amp*) *1 *[34], *pinoid *(*pid*) *1 *[35], and *yellow variegated *(*var*) *2 *[36], were isolated and their mutated genes were determined by PCR or HRM analysis and sequenced using specific primers (see Additional files 1 and 2). The M_3 _seeds of the mutants were harvested and the phenotype of the M_3 _plants was analysed to confirm whether the phenotype of the mutants was inherited.

## Authors' contributions

TA conceived the study, designed the research, coordinated the project and obtained the beam times. YK and TA participated in the design of the molecular genetic analyses. YK, TH, HS, SO, YH, and TA performed C-ion irradiation. YK, TH, HS, YL, and SO conducted mutant screening. YL and SO participated in growing plants. YK, TH, and YL carried out the molecular genetic analyses. YK and TA were primarily responsible for drafting and revising the manuscript with contributions from the co-authors. All authors read and approved the final manuscript.

## Supplementary Material

Additional file 1 **Primers used for HRM**. Click here for file

Additional file 2 **Primers used for other PCR analyses**. Click here for file

## References

[B1] Richardson FC Richardson KK Sequence-dependent formation of alkyl DNA adducts: a review of methods, results, and biological correlates Mutat Res 1990 233 127 138 10.1016/0027-5107(90)90157-Y 2233794

[B2] Greene EA Codomo CA Taylor NE Henikoff JG Till BJ Reynolds SH Enns LC Burtner C Johnson JE Odden AR Comai L Henikoff S Spectrum of chemically induced mutations from a large-scale reverse-genetic screen in *Arabidopsis* Genetics 2003 164 731 740 12807792 10.1093/genetics/164.2.731 PMC1462604

[B3] McCallum CM Comai L Greene EA Henikoff S Targeted screening for induced mutations Nature Biotech 2000 18 455 457 10.1038/74542 10748531

[B4] Wittwer CT Reed GH Gundry CN Vandersteen JG Pryor RJ High-resolution genotyping by amplicon melting analysis using LCGreen Clin Chem 2003 49 853 860 10.1373/49.6.853 12765979

[B5] Till BJ Reynolds SH Weil C Springer N Burtner C Young K Bowers E Codomo CA Enns LC Odden AR Greene EA Comai L Henikoff S Discovery of induced point mutations in maize genes by TILLING BMC Plant Biol 2004 4 12 10.1186/1471-2229-4-12 15282033 PMC512284

[B6] Till BJ Cooper J Tai TH Colowit P Greene EA Henikoff S Comai L Discovery of chemically induced mutations in rice by TILLING BMC Plant Biol 2007 7 19 10.1186/1471-2229-7-19 17428339 PMC1858691

[B7] Cooper JL Till BJ Laport RG Darlow MC Kleffner JM Jamai A El-Mellouki T Liu S Ritchie R Nielsen N Bilyeu KD Meksem K Comai L Henikoff S TILLING to detect induced mutations in soybean BMC Plant Biol 2008 8 9 10.1186/1471-2229-8-9 18218134 PMC2266751

[B8] Cooper JL Till BJ Laport RG Darlow MC Kleffner JM Jamai A El-Mellouki T Liu S Ritchie R Nielsen N Bilyeu KD Meksem K Comai L Henikoff S A modified TILLING approach to detect induced mutations in tetraploid and hexaploid wheat BMC Plant Biol 2009 9 115 10.1186/1471-2229-9-115 19712486 PMC2748083

[B9] Shirley BW Hanley S Goodman HM Effects of ionizing radiation on a plant genome: analysis of two *Arabidopsis transparent testa *mutations Plant Cell 1992 4 333 347 1354004 10.1105/tpc.4.3.333 PMC160133

[B10] Cecchini E Mulligan BJ Covey SN Miner JJ Characterization of gamma irradiation-induced deletion mutations at a selectable locus in *Arabidopsis* Mutation Res 1998 401 199 206 10.1016/S0027-5107(98)00009-8 9639705

[B11] Morita R Kusaba M Iida S Yamaguchi H Nishio T Nishimura M Molecular characterization of mutations induced by gamma irradiation in rice Genes Genet Syst 2009 84 361 370 10.1266/ggs.84.361 20154423

[B12] Abe T Bae CH Ozaki T Wang K Yoshida S Stress tolerant mutants induced by heavy -ion beams Gamma Field Symp 2000 39 45 54

[B13] Tanaka A Shikazono N Hase Y Studies on biological effects of ion beams on lethality, molecular nature of mutation, mutation rate, and spectrum of mutation phenotype for mutation breeding in higher plants J Radiat Res 2010 51 223 233 10.1269/jrr.09143 20505261

[B14] Miyazaki K Suzuki K Iwaki K Kusumi T Abe T Yoshida S Fukui H Flower pigment mutations induced by heavy ion beam irradiation in an inter specific hybrid of Torenia Plant Biotech 2008 23 163 167

[B15] Kanaya T Saito H Hayashi Y Fukunishi N Ryuto H Miyazaki K Kusumi T Abe T Suzuki K Heavy-ion beam-induced sterile mutants of verbena (*Verbena × hybrida*) with an improved flowering habit Plant Biotech 2008 25 91 96 10.5511/plantbiotechnology.25.91

[B16] Ryuto H Fukunishi N Hayashi Y Ichida H Abe T Kase M Yano Y Heavy-ion beam irradiation facility for biological samples in RIKEN Plant Biotech 2008 25 119 122 10.5511/plantbiotechnology.25.119

[B17] Ward JF The complexity of DNA damage: relevance to biological consequences Int J Rad Biol 1994 66 427 432 10.1080/09553009414551401 7983426

[B18] Goodhead DT Molecular and cell models of biological effects of heavy ion radiation Radiat Environ Biophys 1995 34 67 72 10.1007/BF01275208 7652153

[B19] Hoglund E Blomquist E Carlsson J Stenerlow B DNA damage induced by radiation of different linear energy transfer: initial fragmentation Int J Radiat Biol 2000 76 539 547 10.1080/095530000138556 10815635

[B20] Yokota Y Yamada S Hase Y Shikazono N Narumi I Tanaka A Inoue M Initial yields of DNA double-strand breaks and DNA Fragmentation patterns depend on linear energy transfer in tobacco BY-2 protoplasts irradiated with helium, carbon and neon ions Radiat Res 2007 167 94 101 10.1667/RR0701.1 17214518

[B21] Shikazono N Suzuki C Kitamura S Watanabe H Tano S Tanaka A Analysis of mutations induced by carbon ions in *Arabidopsis thaliana* J Exp Bot 2005 56 587 596 10.1093/jxb/eri047 15642718

[B22] Kazama Y Saito H Yamamoto YY Hayashi Y Ichida H Ryuto H Fukunishi N Abe T LET-dependent effects of heavy-ion beam irradiation in *Arabidopsis thaliana* Plant Biotech 2008 25 113 117 10.5511/plantbiotechnology.25.113

[B23] Shitsukawa N Ikari C Shimada S Kitagawa S Sakamoto K Saito H Ryuto H Fukunishi N Abe T Takumi S Nasuda S Murai K The einkorn wheat (*Triticum monococcum*) mutant, maintained vegetative phase, is caused by a deletion in the *VRN1 *gene Genes Genet Syst 2007 82 167 170 10.1266/ggs.82.167 17507783

[B24] Koornneef M Rolff E Spruit CJP Genetic control of light-inhibited hypocotyl elongation in *Arabidopsis thaliana *(L) Heynh Z Pflanzenphysiol 1980 100 147 160

[B25] Reed JW Nagpal P Poole DS Furuya M Chory J Mutations in the gene for the red far-red light receptor phytochrome-B alter cell elongation and physiological responses throughout *Arabidopsis *development Plant Cell 1993 5 147 157 8453299 10.1105/tpc.5.2.147 PMC160258

[B26] Ahmad M Cashmore AR *HY4 *gene of *A. thaliana *encodes a protein with characteristics of a blue-light photoreceptor Nature 1994 366 162 166 10.1038/366162a0 8232555

[B27] Muramoto T Kohchi T Yokota A Hwang IH Goodman HM The *Arabidopsis *photomorphogenic mutant *hy1 *is deficient in phytochrome chromophore biosynthesis as a result of a mutation in a plastid heme oxygenase Plant Cell 1999 11 335 347 10072395 10.1105/tpc.11.3.335 PMC144190

[B28] Kohchi T Mukougawa K Frankenberg N Masuda M Yokota A Lagarias JC The Arabidopsis *HY2 *gene encodes phytochromobilin synthase, a ferredoxin-dependent biliverdin reductase Plant Cell 2001 13 425 436 11226195 10.1105/tpc.13.2.425 PMC102252

[B29] Walker AR Davison PA Bolognesi-Winfield AC James CM Srinivasan N Blundell TL Esch JJ Marks MD Gray JC The *TRANSPARENT TESTA GLABRA1 *locus, which regulates trichome differentiation and anthocyanin biosynthesis in Arabidopsis, encodes a WD40 repeat protein Plant Cell 1999 11 1337 1350 10402433 10.1105/tpc.11.7.1337 PMC144274

[B30] Oppenheimer DG Herman PL Sivakumaran S Esch J Marks MD A *myb *gene required for leaf trichome differentiation in Arabidopsis is expressed in stipules Cell 1991 67 483 493 10.1016/0092-8674(91)90523-2 1934056

[B31] Rerie WG Feldmann KA Marks MD The *GLABRA2 *gene encodes a homeo domain protein required for normal trichome development in *Arabidopsis* Genes Dev 1994 8 1388 1399 10.1101/gad.8.12.1388 7926739

[B32] Bruggemann E Handwerger K Essex C Storz G Analysis of fast neutron-generated mutants at the *Arabidopsis thaliana HY4 *locus Plant J 1996 10 755 760 10.1046/j.1365-313X.1996.10040755.x 8893551

[B33] Shikazono N Tanaka A Watanabe H Tano S Rearrangements of the DNA in carbon ion-induced mutants of *Arabidopsis thaliana* Genetics 2001 157 379 387 11139518 10.1093/genetics/157.1.379 PMC1461491

[B34] Helliwell CA Chin-Atkins AN Wilson IW Chapple R Dennis ES Chaudhury A The Arabidopsis AMP1 gene encodes a putative glutamate carboxypeptidase Plant Cell 2001 13 2115 2125 11549767 10.1105/TPC.010146 PMC139455

[B35] Christensen SK Dagenais N Chory J Weigel D Regulation of auxin response by the protein kinase *PINOID* Cell 2000 100 469 478 10.1016/S0092-8674(00)80682-0 10693763

[B36] Takechi K Sodmergen Murata M Motoyoshi F Sakamoto W The *YELLOW VARIEGATED *(*VAR2*) locus encodes a homologue of FtsH, an ATP-dependent protease in *Arabidopsis* Plant Cell Physiol 2000 41 1334 1346 10.1093/pcp/pcd067 11134419

[B37] Gorbunova V Levy AA How plants make ends meet: DNA double-strand break repair Trends Plant Sci 1999 4 263 269 10.1016/S1360-1385(99)01430-2 10407442

[B38] Koornneef M Dellaert LW van der Veen JH EMS- and radiation-induced mutation frequencies at individual loci in Arabidopsis thaliana (L.) Heynh Mutat Res 1982 93 109 123 10.1016/0027-5107(82)90129-4 7062928

[B39] Naito K Kusaba M Shikazono N Takano T Tanaka A Tanisaka T Nishimura M Transmissible and nontransmissible mutations induced by irradiating *Arabidopsis thaliana *pollen with gamma-rays and carbon ions Genetics 2005 169 881 889 10.1534/genetics.104.033654 15371348 PMC1449103

[B40] Masumura K Kuniya K Kurobe T Fukuoka M Yatagai F Nohmi T Heavy-ion-induced mutations in the gpt delta transgenic mouse: comparison of mutation spectra induced by heavy-ion, X-ray, and gamma-ray radiation Environ Mol Mutagen 2002 40 207 215 10.1002/em.10108 12355555

[B41] Suzuki M Watanabe M Kanai T Kase Y Yatagai F Kato T Matsubara S LET dependence of cell death, mutation induction and chromatin damage in human cells irradiated with accelerated carbon ions Adv Space Res 1996 18 127 136 11538953 10.1016/0273-1177(95)00799-k

[B42] Ottolenghi A Merzagora M Tallone L Durante M Paretzke HG Wilson WE The quality of DNA double-strand breaks: a Monte Carlo simulation of the end-structure of strand breaks produced by protons and alpha particles Radiat Environ Biophys 1995 34 239 244 10.1007/BF01209749 8749062

[B43] Alloni D Campa A Belli M Esposito G Facoetti A Friedland W Liotta M Mariotti L Paretzke HG Ottolenghi A A Monte Carlo study of the radiation quality dependence of DNA fragmentation spectra Radiat Res 2010 173 263 271 10.1667/RR1957.1 20199211

[B44] Blocher D DNA double-strand break repair determines the RBE of alpha-particles Int J Radiat Biol 1988 54 761 771 10.1080/09553008814552201 2902170

[B45] Pastwa E Neumann RD Mezhevaya K Winters TA Repair of radiation-induced DNA double-strand breaks is dependent upon radiation quality and the structural complexity of double-strand breaks Radiat Res 2003 159 251 261 10.1667/0033-7587(2003)159[0251:RORIDD]2.0.CO;2 12537531

[B46] Lobrich M Cooper PK Rydberg B Non-random distribution of DNA double-strand breaks induced by particle irradiation Int J Radiat Biol 1996 70 493 503 10.1080/095530096144680 8947529

[B47] Ichida H Matsuyama T Ryuto H Hayashi Y Fukunishi N Abe T Koba T Molecular characterization of microbial mutations induced by ion beam irradiation Mutat Res 2008 639 101 107 10.1016/j.mrfmmm.2007.10.009 18068202

[B48] Ryuto H Abe T Fukunishi N Kase M Yano Y Heavy-ion beam irradiation system for biological samples in RIKEN J Biomed Nanotech 2006 2 88 93 10.1166/jbn.2006.014

[B49] Scholz M Horowitz Y Dose response of biological systems to low- and high-LET radiation Microdosimetric response of physical and biological systems to low- and high-LET radiations 2006 2006 Oxford, UK: Elsevier 1 73

[B50] Liu YG Mitsukawa N Oosumi T Whittier RF Efficient isolation and mapping of Arabidopsis thaliana T-DNA insert junctions by thermal asymmetric interlaced PCR Plant J 1995 8 457 463 10.1046/j.1365-313X.1995.08030457.x 7550382

